# 2′-*O* Methylation of the Viral mRNA Cap by West Nile Virus Evades Ifit1-Dependent and -Independent Mechanisms of Host Restriction In Vivo

**DOI:** 10.1371/journal.ppat.1002698

**Published:** 2012-05-10

**Authors:** Kristy J. Szretter, Brian P. Daniels, Hyelim Cho, Maria D. Gainey, Wayne M. Yokoyama, Michael Gale, Herbert W. Virgin, Robyn S. Klein, Ganes C. Sen, Michael S. Diamond

**Affiliations:** 1 Department of Medicine, Washington University School of Medicine, St. Louis, Missouri, United Stated of America; 2 Department of Anatomy & Neurobiology, Washington University School of Medicine, St. Louis, Missouri, United Stated of America; 3 Department of Molecular Microbiology, Washington University School of Medicine, St. Louis, Missouri, United Stated of America; 4 Department of Pathology & Immunology, Washington University School of Medicine, St. Louis, Missouri, United Stated of America; 5 Department of Immunology, University of Washington School of Medicine, Seattle, Washington, United States of America; 6 Cleveland Clinic, Lerner Research Institute, Cleveland, Ohio, United States of America; Harvard Medical School, New England Primate Research Center, United States of America

## Abstract

Prior studies have shown that 2′-*O* methyltransferase activity of flaviviruses, coronaviruses, and poxviruses promotes viral evasion of Ifit1, an interferon-stimulated innate immune effector protein. Viruses lacking 2′-*O* methyltransferase activity exhibited attenuation in primary macrophages that was rescued in cells lacking *Ifit1* gene expression. Here, we examined the role of Ifit1 in restricting pathogenesis *in vivo* of wild type WNV (WNV-WT) and a mutant in the NS5 gene (WNV-E218A) lacking 2′-*O* methylation of the 5′ viral RNA cap. While deletion of Ifit1 had marginal effects on WNV-WT pathogenesis, WNV-E218A showed increased replication in peripheral tissues of *Ifit1* ^−/−^ mice after subcutaneous infection, yet this failed to correlate with enhanced infection in the brain or lethality. In comparison, WNV-E218A was virulent after intracranial infection as judged by increased infection in different regions of the central nervous system (CNS) and a greater than 16,000-fold decrease in LD_50_ values in *Ifit1* ^−/−^ compared to wild type mice. *Ex vivo* infection experiments revealed cell-type specific differences in the ability of an Ifit1 deficiency to complement the replication defect of WNV-E218A. In particular, WNV-E218A infection was impaired in both wild type and *Ifit1* ^−/−^ brain microvascular endothelial cells, which are believed to participate in blood-brain barrier (BBB) regulation of virus entry into the CNS. A deficiency of Ifit1 also was associated with increased neuronal death *in vivo*, which was both cell-intrinsic and mediated by immunopathogenic CD8^+^ T cells. Our results suggest that virulent strains of WNV have largely evaded the antiviral effects of Ifit1, and viral mutants lacking 2′-*O* methylation are controlled *in vivo* by Ifit1-dependent and -independent mechanisms in different cell types.

## Author Summary

We recently showed that a West Nile virus (WNV) mutant in NS5 (WNV-E218A) lacking 2′-*O* methyltransferase activity was attenuated in primary macrophages but replicated well in cells lacking type I interferon (IFN) signaling or expression of Ifit1, an IFN-stimulated gene. Here, we follow-up these studies by examining the pathogenesis in *Ifit1^−/−^* mice of WNV-E218A, the mutant virus lacking 2′-*O* methyltransferase activity. Because a deficiency of Ifit1 did not alter pathogenesis of wild type WNV, we conclude that the viral 2′-*O* methyltransferase encoded by NS5 largely overcomes Ifit1-mediated control of infection. In comparison, WNV-E218A showed increased infection in peripheral tissues of *Ifit1* ^−/−^ mice after subcutaneous infection, yet this did not result in enhanced replication in the brain. However, WNV-E218A caused lethal infection when it was directly introduced into the brain. We observed cell-type specific differences in the ability of an Ifit1 deficiency to rescue replication of WNV-E218A; for example, WNV-E218A showed equivalently impaired infection in wild type and *Ifit1* ^−/−^ brain endothelial cells, which potentially allow virus access into the brain. Our results suggest that virulent strains of WNV have evaded the antiviral effects of Ifit1, and mutants lacking 2′-*O* methylation are restricted by cell-type specific Ifit1-dependent and -independent mechanisms.

## Introduction

Type I interferon (IFN) restricts infection of many viruses through cell-intrinsic and cell-extrinsic effects on replication, and by priming adaptive B and T cell responses (reviewed in [1]). Expression of type I IFN after RNA virus infection generally occurs after recognition of viral RNA by pathogen recognition receptors in the cytoplasm (by RIG-I and MDA5) or the endosome (TLR3, TLR7, and TLR8), and initiation of signaling cascades that result in translocation of interferon regulatory factors (IRF-3 and IRF-7) with transcriptional activity (reviewed in [2]). Secreted type I IFN binds to the IFN-αβ receptor (IFNAR) in autocrine and paracrine fashion, activating the Janus kinase and signal transducer and activator of transcription (JAK/STAT) pathway, which induces the expression of hundreds of interferon stimulated genes (ISG) with the potential for antiviral function against a range of viruses [3].

Ifit1 (ISG56, p56) is a highly induced ISG with tetratricopeptide repeats, and a member of an evolutionarily conserved family of proteins that are expressed in response to type I IFN, interleukin-1 (IL-1), tumor necrosis factor alpha (TNF-α) and certain pathogen associated molecular patterns (PAMPs) (reviewed in [4]). In humans, the *IFIT* gene family consists of four members: *IFIT1*, *IFIT2* (ISG54, p54), *IFIT3* (ISG60, p60), and *IFIT5* (ISG58, p58), whereas mice encode three related genes: *Ifit1*, *Ifit2*, and *Ifit3* (ISG49, p49); human IFIT1 and mouse Ifit1 show 53% sequence identity at the amino acid level. Infection and replication of DNA and RNA viruses are potent inducers of *Ifit* family gene expression in many cell types [5]–[7]. Initial studies suggested that human IFIT proteins exerted their antiviral function by inhibiting protein translation through interaction with specific subunits of translation initiation factor eIF3 [8]–[13]. More recent studies have suggested additional inhibitory mechanisms including the control of translation and/or replication of viral RNA lacking 2′-O-methylation of the 5′ cap [14], [15], sequestration of specific viral RNA, including 5′-ppp RNA [16], and direct binding and inhibition of viral proteins [17]. In cell culture, human and mouse IFIT1/Ifit1 reportedly have antiviral activity against several viruses including human papilloma, Sindbis, vesicular stomatitis, and hepatitis C viruses [13], [16], [18]–[20]. In cell culture and mouse models of infection, WNV strongly induces *Ifit1* gene expression in target cells via IFN-dependent and -independent signaling pathways [6], [7].

West Nile Virus (WNV) is an enveloped, single-stranded positive sense RNA virus in the *Flaviviridae* family and an emerging cause of epidemic encephalitis worldwide [21]. Following peripheral infection, WNV replication is thought to occur in subsets of dendritic cells. These cells migrate to and seed draining lymph nodes, resulting in viremia and subsequent infection of visceral organs such as the spleen. By the end of the first week, WNV is largely cleared from peripheral tissues and spreads to the CNS with infection and injury of neurons in the cerebral cortex, hippocampus, brain stem, and spinal cord. Although the exact entry route of WNV into the CNS remains unclear, it has been proposed to enter via retrograde axonal transport from peripheral neurons [22], direct infection of brain microvascular endothelial cells [23], inflammation-induced disruption of BBB integrity [24]–[26], or trafficking of virus-infected leukocytes [27], [28].

We and others have described a WNV mutant with a site-specific substitution in the NS5 gene (WNV-E218A) that abolishes 2′-*O*-methyltransferase activity [29] and attenuates infection in wild type mice and cells [14]; replication of WNV-E218A in primary macrophages, however, was rescued in the absence of IFNAR or Ifit1 [14]. Here, we follow-up on these studies by examining the role of Ifit1 in restricting pathogenesis *in vivo* of wild type WNV (WNV-WT) and WNV-E218A. While Ifit1 had a marginal impact on WNV-WT pathogenesis, replication of WNV-E218A was increased in peripheral tissues of *Ifit1* ^−/−^ mice after subcutaneous infection, yet this failed to result in efficient spread to the brain. In contrast, direct introduction of WNV-E218A into the brain of *Ifit1* ^−/−^ mice was associated with rapid infection of neurons resulting in uniform lethality. *Ex vivo* infection experiments revealed cell-type specific differences in the ability of Ifit1 deficiency to rescue the replication defect of WNV-E218A. Our results suggest that viral mutants lacking 2′-*O* methylation are controlled *in vivo* by *Ifit1*-dependent and -independent mechanisms.

## Results

### WNV-E218A shows enhanced replication but not virulence in *Ifit1* ^−/−^ mice after peripheral infection

**Figure 1:**
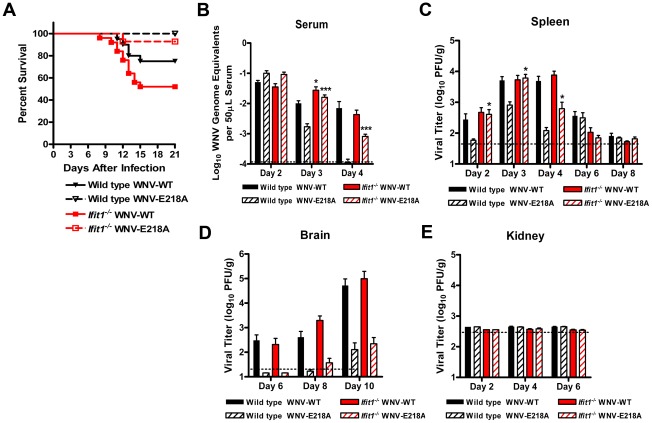
Survival and viral burden analysis after subcutaneous infection of wild type and *Ifit1^−/−^* C57BL/6 mice. A. Nine week-old wild type (*n* = 20) and *Ifit1^−/−^* (*n* = 25) mice were inoculated subcutaneously with WNV-NY (10^2^ PFU, solid lines) or WNV-E218A (10^5^ PFU, dotted lines) and mice were monitored for mortality over 21 days. (**B–D**) WNV tissue burden and spread in mice after subcutaneous infection. *Ifit1^−/−^* and wild type mice were infected with WNV-WT or WNV-E218A via subcutaneous injection in the footpad. At the indicated times post infection, tissues were harvested and analyzed for viral burden by quantitative RT-PCR (**B**) or plaque assay (**C–E**). Data is shown as WNV genome equivalents per 50 µl of serum or PFU per gram of tissue for 10 to 15 mice per time point. Error bars represent standard error of the mean and dotted lines indicate the limit of detection of the assays. Asterisks indicate values that are statistically significant between wild type and *ifit1^−/−^* mice (*, *P*<0.05; ***, *P*<0.0005).

To determine the significance of Ifit1 in restricting WNV infection, and the role of 2′-*O* methylation in this process, we assessed WNV infection of *Ifit1* ^−/−^ mice. We obtained *Ifit1* ^−/−^ C57BL/6 embryonic stem (ES) cells from the trans-NIH Knock-Out Mouse Project (KOMP) consortium and generated mice with a targeted deletion; cells derived from these mice lacked expression of *Ifit1* yet induced normal levels of *Ifit2* (**Figure S1 and S2**). We challenged eight week-old wild type and *Ifit1* ^−/−^ mice via a subcutaneous route with 10^2^ PFU of a virulent strain of WNV-NY (derived from the 385-99 infectious clone; New York, 1999) and compared this to survival after infection with WNV-E218A, a recombinant virus generated from the parent infectious clone with a point mutation in NS5 that abolishes its 2′-*O* methyltransferase activity [14], [29]. *Ifit1* ^−/−^ mice infected with WNV-WT showed a trend towards increased mortality, although this failed to attain statistical significance (52% survival for *Ifit1* ^−/−^ mice compared to 75% survival for wild type mice, *P* = 0.1; **Figure 1A**); thus, in contrast to previous results with other ISGs [30], [31], an absence of *Ifit1* did not markedly enhance pathogenesis of the virulent North American WNV strain. As seen previously [14], the WNV-E218A variant lacking 2′-*O* methylation of its viral RNA cap was highly attenuated in wild type mice, as neither morbidity nor mortality was observed. Somewhat surprisingly, given its rescue in *Ifit1* ^−/−^ macrophages, the WNV-E218A mutant remained attenuated in *Ifit1* ^−/−^ mice, as no disease was observed after subcutaneous infection with 10^2^ or 10^5^ PFU of virus or after infection of younger more vulnerable five week-old mice (**Figure 1A**, and data not shown). This suggests that mechanisms beyond Ifit1-mediated control also contribute to the attenuation of WNV lacking 2′-*O* methylation.

To begin to understand why an absence of *Ifit1* failed to enhance virulence of WNV, mice were infected with WNV-WT or WNV-E218A and viral burden was examined at different days after infection in serum, peripheral organs (spleen and kidney) and the brain. Consistent with the lack of a significant mortality phenotype in *Ifit1* ^−/−^ mice, WNV-WT accumulated to equivalent levels in the blood, spleen, and brain of wild type and *Ifit1* ^−/−^ mice (*P*>0.2), with the exception of a slight (2.7-fold) increase (*P* = 0.04) in serum at day 3 in *Ifit1* ^−/−^ mice (**Figure 1B, C, and D**). Moreover, a deficiency in *Ifit1* did not result in productive infection of the kidney (**Figure 1E**), an organ that is normally resistant to WNV infection in wild type mice yet permissive in animals with defects in type I IFN induction, signaling, or effector functions [31]–[35]. In comparison, despite the absence of mortality after subcutaneous inoculation, WNV-E218A showed enhanced peripheral infection in *Ifit1* ^−/−^ mice and accumulated in the serum and spleen to comparable levels observed in wild type mice infected with WNV-WT (*P*<0.05, comparison of WNV-E218A between wild type and *Ifit1* ^−/−^ mice; **Figure 1B and C**). So why did WNV-E218A fail to cause mortality after subcutaneous infection? Viral burden analysis in the brain revealed markedly lower (14 to 448-fold, *P*<0.01) titers in the brains of wild type and *Ifit1* ^−/−^ mice infected with WNV-E218A at days 6, 8, and 10 after infection (**Figure 1D**). Thus, an absence of *Ifit1* largely restored infection of the 2′-*O* methylation mutant WNV in peripheral tissues but this was not associated with complementation of replication defects in the brain, likely explaining the attenuated disease phenotype of the WNV-E218A mutant in *Ifit1* ^−/−^ mice.

### A deficiency of Ifit1 rescues virulence of WNV-E218A in the CNS

**Figure 2:**
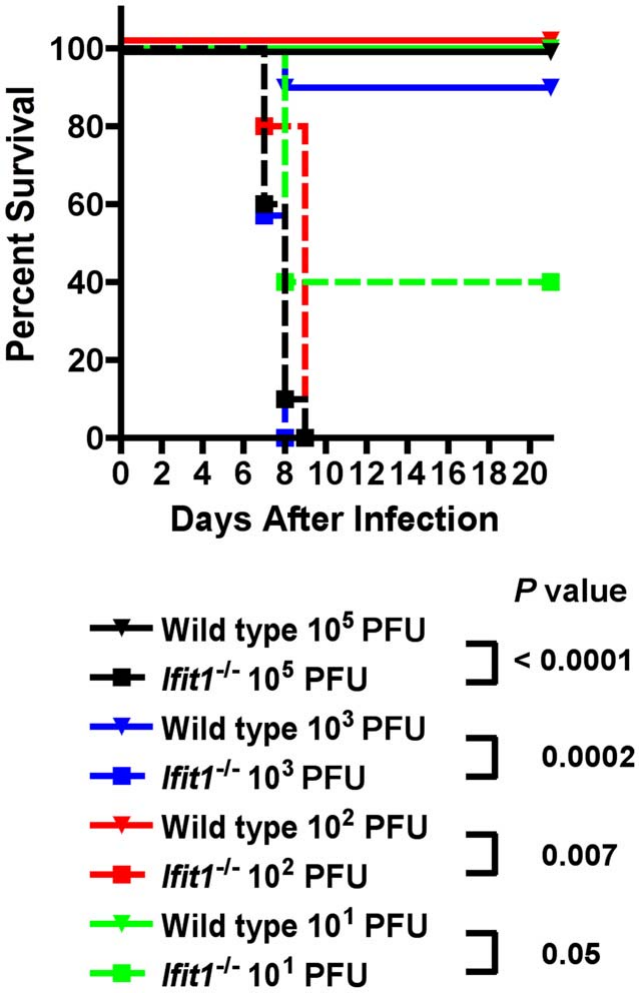
*Ifit1^−/−^* mice are more susceptible to WNV-E218A infection by an intracranial inoculation. Nine week-old wild type (*n* = 4 to 10 per dose) and *Ifit1^−/−^* (*n* = 5 to 10 per dose) mice were inoculated via an intracranial route with serial 10-fold dilutions of WNV-E218A and monitored for 21 days for mortality. The *P* values for specific comparisons of mortality are indicated.

As the subcutaneous infection experiments suggested tissue specific restriction of WNV-E218A by Ifit1, we hypothesized that the 2′-*O* methylation mutant might replicate less efficiently in the central nervous system (CNS), which would explain the attenuated phenotype. To begin to address this, we performed a lethal dose (LD_50_) analysis after intracranial infection of WNV-E218A in wild type and *Ifit1* ^−/−^ mice. While the intracranial infection model is not directly relevant to WNV disease pathogenesis, it allows for examination of viral infection in neurons directly and bypasses barriers associated with neuroinvasion. Notably, in wild type mice, the LD_50_ of WNV-E218A was greater than 10^5^ PFU, as no lethality was observed at even the highest dose tested (**Figure 2**). Remarkably, in *Ifit1* ^−/−^ mice, the virulence of WNV-E218A was restored via the intracranial route with an LD_50_ of 6 PFU (∼16,600-fold lower). In comparison, no appreciable difference of LD_50_ for WNV-WT was observed between wild type and *Ifit1* ^−/−^ mice (4 versus 6 PFU, data not shown). Thus, the defect in neurovirulence in *Ifit1* ^−/−^ mice that was observed after peripheral infection of WNV-E218A was not due to an inherent inability of the 2′-*O* methylation mutant virus to cause disease in the brain.

**Figure 3:**
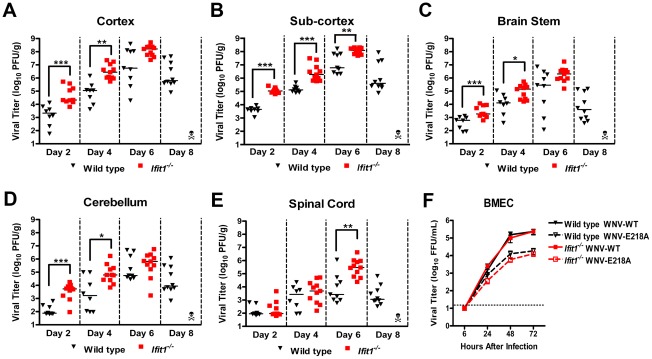
WNV-E218A replication in regions of the CNS of *Ifit1^−/−^* mice after intracranial infection. (**A–E**) *Ifit1^−/−^* or wild type mice were infected with 10^5^ PFU of WNV-E218A via intracranial injection. At the indicated times after infection, (**A**) cerebral cortex, (**B**) sub-cortex, (**C**) brain stem, (**D**) cerebellum, and (**E**) spinal cord were harvested and analyzed for viral burden by plaque assay on BHK21-15 cells. Data is shown as PFU per gram of tissue from 8 to 11 mice per time point. (**F**) Wild type or *Ifit1^−/−^* BMEC were infected at an MOI of 0.01 with WNV-WT or WNV-E218A, and virus yield was titrated at the indicated times by focus forming assay. Values are an average of triplicate samples generated from three independent experiments. Solid lines represent the median viral titer, error bars represent the standard error of the mean, and dotted lines indicate the limit of detection of the respective assays. Asterisks indicate values that are statistically significant between wild type and *Ifit1^−/−^* mice (*, *P*<0.05; **, *P*<0.005; ***, *P*<0.0005).

To extend these findings, we performed plaque assays on tissue homogenates isolated from different regions of the central nervous system (CNS). Wild type and *Ifit1* ^−/−^ mice were infected with WNV-E218A directly into the brain via an intracranial route and viral burden in the cerebral cortex, sub-cortex, brain stem, cerebellum, and spinal cord was measured on days 2, 4, 6, and 8 after infection. At day 2 after intracranial infection, higher WNV-E218A titers were measured in the cortex (35-fold, *P* = 0.0002), sub-cortex (28-fold, *P*<0.0001), brain stem (7-fold, *P* = 0.0009), and cerebellum (31-fold, *P* = 0.0003) in *Ifit1^−/−^* mice compared to wild type mice (**Figure 3A, B, C, and D**). Analogously, higher levels of virus (5 to 38-fold (*P*<0.02)) were measured in these brain regions at day 4 after infection. By day 6, however, no difference in viral burden was detected in cortex, brainstem and cerebellum (*P*>0.09), although WNV-E218A was 3-fold higher (*P* = 0.008) in the sub-cortex and 6-fold higher (*P* = 0.005) in the spinal cord of *ifit1^−/−^* mice (**Figure 3E**). These results demonstrate that a deficiency of Ifit1 enhanced the ability of WNV-E218A to replicate and spread in CNS tissues. Importantly, virus harvested from brain tissues of *Ifit1* ^−/−^ mice at day 6 retained the WNV-E218A mutation as judged by direct sequencing of plaque-purified isolates (data not shown).

While pathogenesis in the CNS was restored when WNV-E218A was directly inoculated into the brain of *Ifit1* ^−/−^ mice, this virus still replicated to relatively high titer in the brains of wild type mice yet failed to induce lethality. To investigate the disparity between the absence of a lethality phenotype and high viral burden data in wild type mice, we assessed WNV-E218A replication on day 8 after intracranial infection, a day by which all *ifit1^−/−^* mice had succumbed to infection. While WNV-E218A replicated to high levels in most regions of the CNS on day 6, we observed a rapid clearance phase with 3 to 36-fold reduced viral titers (*P*<0.05) on day 8 in wild type mice (**Figure 3A, B, C, and D**).

### Ifit1 restricts replication of WNV lacking 2′-*O* methylation in subsets of primary cells

**Figure 4:**
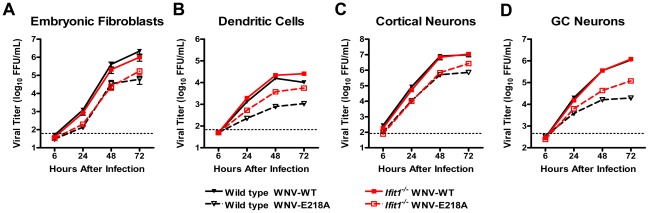
Ifit1 restricts viruses lacking 2′-*O* methylation of the viral mRNA cap infection in some but not all primary cells. (**A**) MEF and (**C**) CN were generated from embryos of wild type and *Ifit1^−/−^*mice, (**D**) GCN were generated from neonatal wild type and *Ifit1^−/−^*mice, and (**B**) DC were derived from bone marrow of adult wild type and *Ifit1^−/−^*mice. Primary cells were infected with (**A–D**) WNV-WT and WNV-E218A at an MOI of 0.01 and virus yield was titrated at the indicated times by focus forming assay on Vero cells. Values are an average of triplicate samples generated from at least three independent experiments. The dotted line represents the limit of detection for the assay. Statistically significant differences are described in the text of the Results.

Given the viral replication phenotype *in vivo*, we hypothesized that Ifit1 differentially mediated antiviral actions within specific cell types. To evaluate this, we compared multi-step growth kinetics of WNV-WT and WNV-E218A in several wild type and *Ifit1^−/−^* primary cells including fibroblasts (MEF), dendritic cells (DC), and neurons from the neocortex (cortical neurons, CN) or hindbrain (cerebellar granular cell neurons, GCN). Initial studies established that none of the primary cells deficient in *Ifit1* sustained enhanced infection with WNV-WT compared to wild type cells (**Figure 4A–D**, *P*>0.1) at any of the time points examined. In comparison, WNV-E218A replicated less efficiently (4 to 19-fold, *P*<0.05) than WNV-WT in wild type MEF, DC, CN, and GCN (**Figure 4A–D**). This defect was partially restored in *Ifit1* ^−/−^ DC (5.2-fold increase, *P*<0.001), CN (4-fold increase at 72 hours, *P*<0.001), and GCN (6.1-fold increase, *P*<0.003) but not substantially altered in MEF. These experiments suggest that flavivirus mutants lacking 2′-*O* methylation are growth inhibited to varying degrees in distinct primary cells, and this occurs through Ifit1-dependent and independent mechanisms. The cell type-specific differences, however, were not due to variation in induction of Ifit1, as the gene was expressed highly in all cells after viral infection (**Figure S2**).

### A deficiency of Ifit1 fails to restore WNV-E218A replication in brain endothelial cells

While WNV-E218A replicated in the brains of *Ifit1^−/−^* mice after direct intracranial inoculation, it failed to sustain high-level infection in the CNS or cause lethality following subcutaneous inoculation, despite achieving near normal titers in the serum and spleen. We hypothesized that WNV-E218A in particular, might have impaired neuroinvasive potential, which was not rescued by a deficiency in Ifit1. Brain microvascular endothelial cells (BMEC) have been hypothesized to participate in the entry of WNV into the CNS, possibly through direct infection or altered barrier functions secondary to systemic inflammation [23]–[26]. To assess whether WNV-E218A had defects in infection of endothelial cells, multi-step viral growth kinetics were performed in BMEC derived from wild type and *Ifit1^−/−^* mice. Notably, WNV-WT replicated to significantly higher levels (6 to 20-fold at 48 and 72 hours, *P*<0.02) than WNV-E218A in wild type BMEC, and this difference was not complemented by a deficiency of Ifit1 (**Figure 3F**); thus, WNV-E218A showed a growth defect in BMEC independent of Ifit1 expression. In comparison, we observed no difference or significant levels of positive strand viral RNA in blood leukocytes at days 3 after infection of WNV-WT or WNV-E218A in *Ifit1* ^−/−^ mice (data not shown). Moreover, no differences in accumulation of intravascular proinflammatory cytokines (IL1-β, IL-6, IFN-γ, and TNF-α) were observed in *Ifit1* ^−/−^ mice infected with WNV-WT or WNV-E218A at days 3 and 4 (**Figure S3**, and data not shown), time points that are believed critical for cytokine-induced modulation of BBB permeability [26]. These observations suggest that the attenuated clinical phenotype of WNV-E218A after subcutaneous infection of wild type or *Ifit1* ^−/−^ mice may be attributed in part to its inability to efficiently infect CNS endothelial cells.

### An absence of Ifit1 leads to immune- and virus-induced pathology after intracranial infection with WNV-E218A

**Figure 5:**
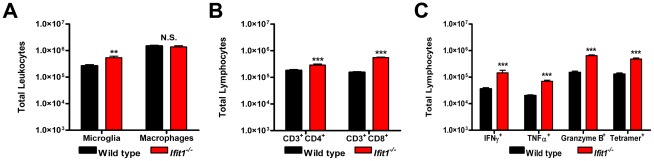
Leukocyte accumulation in the CNS after WNV-E218A infection by an intracranial route. Wild type and *Ifit1^−/−^* mice were inoculated with 10^5^ PFU of WNV-E218A by intracranial injection. (**A–C**) Brains were harvested on day 6 after infection, and leukocytes were isolated by percoll gradient centrifugation. The total number of (**A**) microglia (CD11b^high^/CD45^low^) and macrophages (CD11b^high^/CD45^high^), or (**B**) CD3^+^CD4^+^ or CD3^+^CD8^+^ T cells was determined by multiplying the percentage of each cell population by the total cell count. (**C**) Brain leukocytes were stained with a D^b^-NS4b tetramer or stimulated *ex vivo* with a D^b^-restricted NS4B WNV peptide and then stained for CD3, CD8, granzyme B, IFN-γ or TNF-α, and analyzed by flow cytometry. Data represent the average of at least two independent experiments with 4 to 7 mice per experiment (*n* = 8 to 14). Asterisks indicate values that are statistically significant between wild type and *Ifit1^−/−^* mice (**, *P*<0.005; ***, *P*<0.0005).

To further understand the clinical phenotype of WNV-E218A in *Ifit1* ^−/−^ mice after intracranial infection, we assessed inflammatory cell accumulation in the brains. At day 6 after intracranial infection with WNV-E218A, leukocytes were isolated from brains of wild type and *ifit1^−/−^* mice and analyzed by flow cytometry. Notably, higher numbers of activated CD11b^high^ CD45^low^ microglia (2-fold, **Figure 5A**, *P* = 0.005), CD3^+^CD4^+^ T cells (2-fold, **Figure 5B**, *P* = 0.0009), and CD3^+^CD8^+^ T cells (4-fold, **Figure 5B**, *P*<0.0001) were detected in the brains of *ifit1^−/−^* mice, although no difference in the number of CD11b^high^ CD45^high^ macrophages was observed (**Figure 5A**, *P*>0.4). The CD8^+^ T cell response in the brain was WNV-specific, as higher numbers of cells expressing IFN-γ (4-fold, **Figure 5C**, *P*<0.0001), TNF-α (3-fold, **Figure 5C**, *P*<0.0001), or granzyme B (4-fold, **Figure 5C**, *P*<0.0001) were detected after *ex vivo* restimulation with a D^b^-restricted WNV NS4B peptide [36], and elevated numbers of D^b^-NS4B tetramer-positive cells also were measured (4-fold, **Figure 5C**, *P*<0.0001). These results confirm that wild type mice mount a robust cellular immune response to WNV-E218A, which likely contributes to viral clearance observed at day 8 (see **Figure 3**). In comparison, a deficiency of Ifit1 was associated with increased infection by WNV-E218A, which likely secondarily resulted in enhanced recruitment of lymphocytes to the CNS. This response in *Ifit1* ^−/−^ mice nonetheless failed to control infection and/or contributed to lethality by inducing immunopathology.

**Figure 6:**
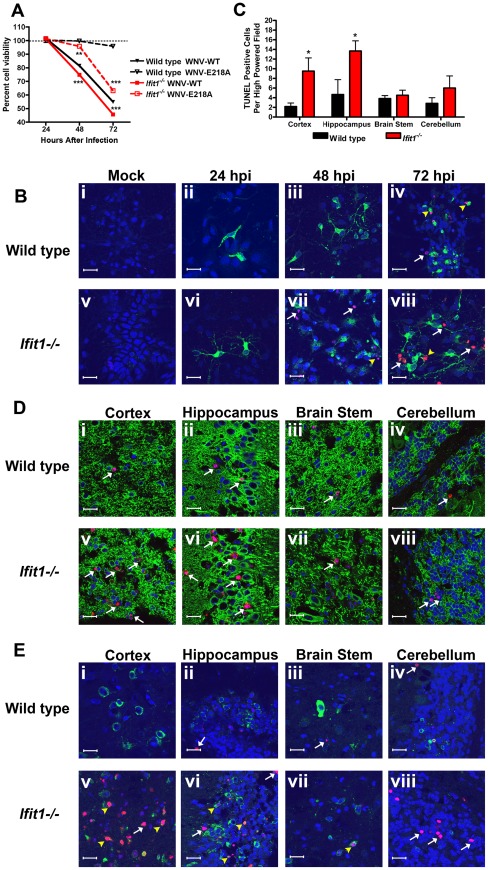
Ifit1 protects against neuronal death during WNV infection. (**A**) CN were infected at an MOI 0.01 with WNV-WT or WNV-E218A and cell viability evaluated at indicated times using a CellTiter-Glo commercial assay. Data represents the percentage of viable cells normalized to mock-infected neurons at each time point. (**B**) CN were seeded on coverslips and infected at an MOI 0.01 with WNV-E218A. At the indicated time points, coverslips were fixed and co-stained for WNV antigen (*green*), TUNEL (*red*) and the nuclear stain ToPro-3 (*blue*). Representative images of ten high-powered fields from at least three separate coverslips are shown. (**C–E**) Wild type and *Ifit1^−/−^* mice were infected with 10^5^ PFU of WNV-E218A via intracranial injection. At day 6, tissues were harvested and processed as described in the Methods. (**C**) The number of TUNEL-positive cells was quantified from three high-powered fields per brain region per mouse from four independent mice. Fixed, frozen section from the (**D: i and v**) cerebral cortex, (**D: ii and vi**) hippocampus, (**D: iii and vii**) brain stem, and (**D: iv and viii**) cerebellum were co-stained for the MAP-2 neuronal antigen (*green*), TUNEL (*red*) and the nuclear stain ToPro-3 (*blue*). Fixed, frozen section from the (**E: i and v**) cerebral cortex, (**E: ii and vi**) hippocampus, (**E: iii and vii**) brain stem, and (**E: iv and viii**) cerebellum were co-stained for the WNV antigen (*green*), TUNEL (*red*) and the nuclear stain ToPro-3 (*blue*). Images are representative of 4 to 6 mice. White arrows indicate TUNEL-positive neurons, yellow arrowheads indicate TUNEL-positive and WNV antigen-positive cells and bars indicate 20 µM. Asterisks indicate values that are statistically significant between wild type and *Ifit1^−/−^* mice (*, *P*<0.05; **, *P*<0.005, ***, *P*<0.0001).

Lethality from neurotropic viruses, such as WNV can be attributed to both cell-intrinsic and cell-extrinsic mechanisms [37], [38]. We speculated that part of the clinical phenotype after intracranial infection of WNV-E218A in *Ifit1* ^−/−^ mice was due to accelerated neuronal death. To address this idea using an *in vitro* model, we assessed the effect of Ifit1 on WNV-induced cell death in CN. CN from wild type and *Ifit1^−/−^* mice were infected (MOI of 0.01) with WNV-WT or WNV-E218A, and cell viability was assessed initially by measuring levels of intracellular ATP. At 48 and 72 hours after infection, *Ifit1^−/−^* CN infected with WNV-WT showed lower viability compared to wild type CN (48 hours: 83.4% viable wild type cells versus 73.3% viable *Ifit1* ^−/−^ cells, *P*<0.0001; and 72 hours: 58.8% viable wild type cells versus 45.2% viable *Ifit1* ^−/−^ cells, *P*<0.0001) (**Figure 6A**); this effect, however, was not associated with differences in viral replication between wild type and *Ifit1* ^−/−^ CN (see **Figure 4C**). In comparison, *Ifit1^−/−^* CN infected with WNV-E218A showed slightly decreased viability at 48 hours (94.8% in *Ifit1* ^−/−^ CN versus 98.4% in wild type CN, *P* = 0.005) and significantly lower viability at 72 hours (61.4% in *Ifit1* ^−/−^ CN versus 96.4% in wild type CN, *P*<0.0001) (**Figure 6A**), although part of this phenotype may be explained by the 4-fold increase in replication in CN at 72 hours (see **Figure 4C**). Consistent with these results, in confocal microscopy experiments with WNV-E218A-infected primary CN, we observed enhanced TUNEL staining in *Ifit1* ^−/−^compared to wild type cells at 48 and 72 hours despite similar levels of viral antigen (**Figure 6B**). While some cells co-stained for TUNEL and WNV antigen (*white arrows*), the majority of TUNEL-positive neurons appeared negative for WNV antigen yet were in proximity to clusters of infected cells (*yellow arrowheads*). Although more investigation is warranted, these neurons may have undergone cell death after WNV infection and leaked cytoplasmic viral antigen or were injured in a bystander fashion from excitotoxic amino acids, peptides, or proteins [39] that were released by adjacently infected cells.

To assess whether a corresponding phenotype was observed *in vivo*, we stained fixed frozen sections from brains of wild type and *Ifit1^−/−^* mice infected via the intracranial route with WNV-E218A for qualitative and quantitative analysis of cell death via TUNEL assay. At day 6 after infection, a time point when relatively similar amounts of infectious virus was detected in several regions of the brain of wild type and *Ifit1^−/−^* mice (see **Figure 3**), we observed markedly increased numbers of TUNEL-positive neurons in the cerebral cortex (**Figure 6C, and 6D: i** versus **6D: v**, *P* = 0.02) and hippocampus (**Figure 6C, and 6D: ii** versus **6D: vi**, *P* = 0.04) of *Ifit1* ^−/−^ mice, although this pattern was not seen in the brain stem (**Figure 6C, and 6D: iii** versus **6D: vii**) or cerebellum (**Figure 6C, and 6D: iv** versus **6D: viii**). Analysis of adjacent sections that were co-stained for WNV antigen showed similar findings, with an increased number of TUNEL-positive cells in *Ifit1* ^−/−^ mice in the background of similar number of WNV-E218A-infected cells (**Figure 6E: i–viii**). Again, and analogous to results with primary CN (**Figure 6B**), there was a noticeable disparity in TUNEL-positive and WNV-positive cells. Overall, subsets of *Ifit1* ^−/−^ neurons appear more susceptible to WNV-E218A infection and cell death in culture and *in vivo*.

**Figure 7:**
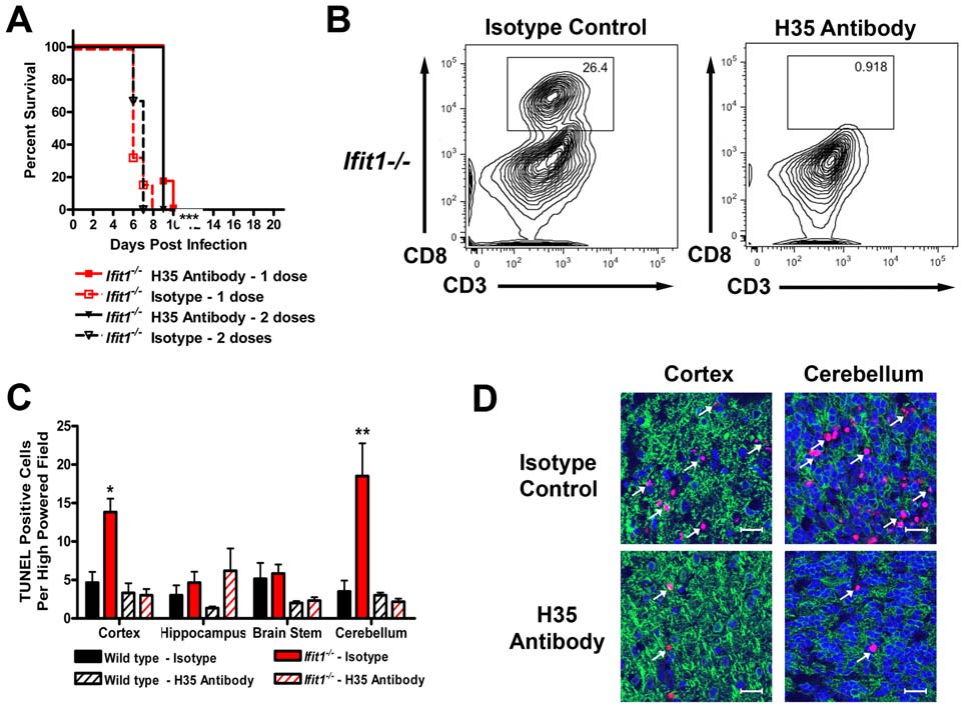
CD8^+^ T cells are pathogenic following WNV-E218A infection. Nine week-old *Ifit1^−/−^* (*n* = 6 to 12) mice were injected via intraperitoneal route with 450 µg of H35 CD8β-depleting antibody or an isotype control one day prior to intracranial inoculation with 10^5^ PFU of WNV-E218A. A subset of mice received a second dose of antibody on day 5 after infection. (**A**) Survival was monitored daily. The difference in mean time to death was statistically significant between isotype and H35 MAb treated *Ifit1^−/−^* mice (*P*<0.0001). (**B**) Representative dot plots illustrating depletion of CD8^+^ T cells in the brains of *Ifit1^−/−^* mice after H35 MAb administration. (**C**) At day 6, tissues were harvested and processed as described in the Methods. Fixed, frozen sections were co-stained for the MAP-2 neuronal antigen (*green*), TUNEL (*red*) and the nuclear stain ToPro-3 (*blue*). The number of TUNEL-positive cells was quantified from three high-powered fields per brain region per mouse from four independent mice. Asterisks indicate values that are statistically significant between H35 MAb and isotype control antibody treated *Ifit1^−/−^* mice (*, *P*<0.05; **, *P*<0.005). (**D**) Representative images of the cerebral cortex and cerebellum from H35 MAb or isotype control antibody treated *Ifit1^−/−^* mice. White arrows indicate TUNEL-positive neurons, and bars indicate 20 µM.

To address whether infiltrating cytotoxic T lymphocytes independently contributed to the lethality phenotype in *Ifit1^−/−^* mice infected with WNV-E218A, we administered a CD8-β-depleting antibody (H35 clone, rat IgG2b) or isotype control to *Ifit1^−/−^* mice by peripheral injection one day prior to challenge of mice with WNV-E218A via an intracranial route. Although all *Ifit1^−/−^* mice succumbed to infection regardless of treatment, depletion of CD8^+^ T cells delayed the mean time to death by 3 days (*P*<0.001, **Figure 7A and B**), suggesting a pathologic effect of these cells in *Ifit1^−/−^* mice. In comparison, no deleterious clinical effect was observed after CD8^+^ T cell depletion of wild type mice infected with WNV-E218A (data not shown). To corroborate these results, we depleted CD8^+^ T cells immediately prior to infection of wild type or *Ifit1* ^−/−^ mice with WNV-E218A, prepared frozen sections from different brain regions at day 6, and quantitated the number of TUNEL-positive cells by confocal microscopy. Consistent with the survival data, depletion of CD8^+^ T cells reduced the number of TUNEL-positive neurons in the cerebral cortex, brain stem, and cerebellum of *Ifit1* ^−/−^ mice (*P*<0.02, **Figure 7C and D**). Nonetheless, there was a limit to the protective effects of CD8^+^ T cell depletion, as additional doses of the H35 antibody failed to prolong survival beyond that observed with a single dose (**Figure 7A**). These data confirm that CD8^+^ T cells have the potential to cause immunopathology associated with WNV infection in the brain [40], and suggest that the accelerated neuronal injury observed in *Ifit1* ^−/−^ mice after WNV-E218A infection is caused in part, by CD8^+^ T cells.

## Discussion

Here, we generated *Ifit1^−/−^* mice and established the role of Ifit1 protein in restricting pathogenesis of wild type WNV and a mutant (NS5-E218A) lacking 2′-*O* methyation of the 5′ cap of its viral RNA. For the virulent wild type strain of WNV, a deficiency of Ifit1 had little impact on infectivity or pathogenesis, as no significant increase in lethality, tissue viral burden, or replication in primary cells was observed. Thus, Ifit1 does not have a dominant role in restricting infection or pathogenesis of wild type WNV, which is consistent with the hypothesis that 2′-*O* methylation of the 5′ viral RNA cap by the viral methyltransferase (NS5) effectively subverts innate host antiviral responses through escape of Ifit1-mediated suppression [14]. In comparison, a deficiency of Ifit1 enhanced the virulence of the attenuated WNV-E218A mutant lacking 2′-*O*-methyation. While infection of wild type mice with WNV-E218A via the subcutaneous route resulted in little apparent disease even with doses up to 10^5^ PFU, *Ifit1^−/−^* mice showed enhanced susceptibility to WNV-E218A with elevated viral burden in serum and spleen, although this failed to result in efficient spread to the brain or cause severe disease. *Ex vivo* infection experiments revealed impaired infectivity of WNV-E218A in both wild type and *Ifit1* ^−/−^ BMEC, which are a major component of the BBB and are hypothesized to contribute to WNV entry into the CNS [23]. Thus, the failure of WNV-E218A to spread to the brain after peripheral inoculation could be due in part, to an Ifit1-independent restriction in BBB infection and virus crossing.

Although Ifit1 is highly expressed in multiple areas of the brain after WNV infection [7], and is thus in position to restrict infection of WNV-E218A, the attenuated phenotype after peripheral infection of *Ifit1* ^−/−^ mice still could have been due to an intrinsic inability to replicate in target neurons of the CNS. Against this, direct inoculation of WNV-E218A into the brain in *Ifit1^−/−^* mice resulted in enhanced replication, inflammation, and neuronal injury, which resulted in lethal infection with an LD_50_ of 6 PFU; in comparison, no lethality was observed in wild type mice after direct intracranial inoculation of even high (10^5^ PFU) doses of WNV-E218A, although high levels (10^5^ to 10^7^ PFU/g) of virus accumulated in multiple regions of the brain. Our studies suggest that lethal phenotype after WNV-E218A infection by the intracranial route in *Ifit1* ^−/−^ mice was due to both cell-intrinsic and extrinsic mechanisms. WNV-infected *Ifit1* ^−/−^ CN were less viable following infection *in vitro*, and the increased viral burden in the brain elicited greater numbers of infiltrating lymphocytes, including CD8^+^ T cells that appeared to contribute to pathogenesis by WNV-E218A. Taken together, our results show that viral mutants lacking 2′-*O* methylation are controlled *in vivo* in part, by Ifit1-dependent mechanisms, and suggest a possible route for WNV neuroinvasion in the CNS.

Our *ex vivo* infection experiments revealed cell-type specific differences in the ability of a deficiency of Ifit1 to rescue the replication defect of WNV-E218A. In prior studies, we showed that *Ifit1* ^−/−^ but not *Ifit2* ^−/−^ macrophages supported almost wild type levels of replication of flaviviruses (WNV-E218A) and coronaviruses (mouse hepatitis virus (MHV)-D130A) lacking 2′-*O* methylation of the viral RNA cap [14], [15]. Here, we observed a range of Ifit1-dependent and -independent phenotypes for restricting replication of WNV-E218A; in no instance, however, did we observe an increase in replication of WNV-WT in any of the *Ifit1* ^−/−^ primary cells examined. Whereas *Ifit1* ^−/−^ DC and GCN sustained enhanced infection of WNV-E218A, *Ifit1* ^−/−^ MEF and CN showed smaller increases in infection, and as mentioned above, no increase in WNV-E218A replication was observed in *Ifit1* ^−/−^ BMEC. A similar pattern was observed with the MHV-D130A 2′-*O* methylation mutant virus, as *Ifit1* ^−/−^ GCN and DC supported enhanced replication but *Ifit1* ^−/−^ CN did not (K. Szretter and M. Diamond, unpublished results). Thus, our cell culture data suggests that viruses lacking 2′-*O* methylation are restricted in a cell-type specific manner by Ifit1-dependent and -independent mechanisms. While a prior study with MHV suggested that viruses lacking 2′-*O* methylation also may be recognized preferentially by the RNA sensor MDA5 [15] resulting in enhanced type I IFN secretion, we did not observe this phenotype with WNV-E218A; we saw no significant restoration in infectivity of WNV-E218A in several different *Mda5* ^−/−^ primary cells and no rescue of virulence in *Mda5* ^−/−^ mice (H. Lazear and M. Diamond, unpublished results). Thus, the identity and biochemical basis of the Ifit1-independent control pathway against WNV-E218A remains unknown, although other gene family members (mouse *Ifit2* or *Ifit3*) are candidates under study.

Despite its robust induction after viral infection or exposure of cells to type I IFN [41]–[43], Ifit1 did not contribute significantly to the control of wild type WNV infection *in vivo*. Similarly, we observed no difference in clinical phenotype between wild type and *Ifit1* ^−/−^ mice after infection with encephalomyocarditis (EMCV), a picornavirus that has a viral protein (VPg) covalently bound to the 5′ end of its positive-strand genomic RNA (V. Fensterl and G. Sen, unpublished results). These results were somewhat surprising, as Ifit1 has been proposed to have antiviral functions beyond its capacity to inhibit infection of viruses lacking 2′-*O*-methylation of the viral mRNA cap. Human or mouse Ifit1 reportedly have antiviral activity in cell culture against human papilloma, Sindbis, Rift valley fever, and hepatitis C viruses [13], [16], [18]–[20], and *in vivo* against vesicular stomatitis virus [16]. While initial studies suggested that Ifit proteins exerted their antiviral function by inhibiting protein translation [8]–[13], additional inhibitory mechanisms have been proposed including sequestration of viral RNA replication intermediates, including 5′-ppp RNA [16], and direct binding and inhibition of viral proteins [17]. While WNV and other flaviviruses have capped genomes at the 5′ end of the infectious positive RNA strand, in theory, Ifit1 could recognize other viral derived RNA with free 5′-ppp including the negative strand RNA replicative intermediate or sfRNA [44], [45]. However, our results showing little difference in replication phenotype for WNV-WT in *Ifit1* ^−/−^ mice and cells suggest that recognition of 5′-ppp RNA is not critical for host control of WNV. Alternatively, a gain-of-function phenotype may not have been observed in *Ifit1* ^−/−^ mice after WNV-WT infection because multiple ISG function in concert to inhibit viral replication *in vivo*, such that targeted deletion of a single gene (e.g. Ifit1) with relatively modest effects results in a minimal phenotype. Indeed, cell culture studies have shown that several different ISG have inhibitory activity against a given family of viruses [3], [19], [46].

The BBB poses a physical obstacle for pathogen entry into the brain through its endothelial cell tight junctions and astrocyte foot processes. Although the exact route of WNV entry into the CNS remains unclear, WNV may enter via axonal spread [22], direct infection of endothelial cells [23], inflammation-induced disruption of BBB integrity [24]–[26] or possibly through transport within trafficking hematopoietic cells, such as neutrophils [28]. Our peripheral and intracranial infection studies with WNV-E218A in *Ifit1* ^−/−^ mice are more consistent with a model in which direct infection of endothelial cells contributes to CNS spread. However, by immunohistochemistry we did not detect WNV antigen in brain endothelial cell from wild type or *Ifit1* ^−/−^ mice (K. Szretter and M. Diamond, unpublished results); this could reflect the ∼100-fold lower level of virus accumulation in BMEC relative to neurons, which may fall below the limit of current histological detection methods. Although WNV-E218A readily infected neurons after direct intracranial infection of *Ifit1* ^−/−^ mice, little replication in the brain was observed after subcutaneous inoculation despite relatively normal levels of viremia, and this finding correlated with an Ifit1-independent mechanism of control in BMEC *ex vivo*. We also cannot exclude the possibility of a transient difference in BBB permeability of *Ifit1* ^−/−^ mice induced specifically by peripheral infection of WNV-WT but not WNV-E218A. Indeed, a recent study suggested that Ifit1 may act a negative regulator of virus-triggered cellular antiviral responses through its binding to STING and disruption of interaction with IPS-1 and TBK1 [47]; *Ifit1* ^−/−^ mice after peripheral infection of WNV-WT and WNV-E218A should therefore have increased inflammation compared to wild type mice. However, we failed to observe significant differences in pro-inflammatory cytokine production in wild type and *Ifit1* ^−/−^ mice after WNV infection. While we did observe increased inflammatory infiltrates in the brains of *Ifit1* ^−/−^ mice infected with WNV-E218A via an intracranial route, this likely was an indirect effect of the enhanced virus replication in neurons, which induces chemokine production that facilitates leukocyte infiltration [48], [49].

An absence of Ifit1 was associated with increased neuronal injury and death *in vivo* and this likely occurred through both cell-intrinsic and cell-extrinsic mechanisms. Infection experiments in primary CN showed that cell viability was compromised more rapidly in *Ifit1* ^−/−^ compared to wild type cells after WNV-WT or WNV-E218A infection, despite relatively equivalent levels of viral production in the supernatant. The mechanistic basis for this remains uncertain although a recent study suggested that expression of IFIT2 modulates apoptosis [50]. *In vivo*, after intracranial infection of WNV-E218A, we observed significantly more TUNEL-positive neurons in *Ifit1* ^−/−^ compared to wild type mice, and this occurred in a region-specific manner. While some of this phenotype was likely due to the enhanced virus replication in *Ifit1* ^−/−^ mice, depletion of CD8^+^ T cells resulted in prolonged survival, suggesting a contribution of immune cell mediated injury. While prior studies have defined a protective role for CD8^+^ T cells after peripheral infection by virulent strains of WNV [36], [51]–[53], our findings with WNV-E218A are more consistent with a report showing immunopathogenic CD8^+^ T cells after infection with an attenuated WNV strain [40]. Likely, for virulent strains of WNV, CD8^+^ T cell-dependent injury of a relatively small number of infected neurons outweighs the risk of widespread cell death induced directly by the virus.

In summary, our results show that Ifit1 restricts infection *in vivo* of WNV lacking 2′-*O* methylation of the 5′ cap structure, as targeted *Ifit1* gene deletion was associated with enhanced replication of WNV-E218A in mice. Because a deficiency of Ifit1 did not significantly alter pathogenesis of WNV-WT, we conclude that 2′-*O* methylation of the 5′ viral RNA cap by the NS5 largely overcomes Ifit1-mediated restriction of infection. Based on tissue and region-specific effects *in vivo*, and infection of primary cells *ex vivo*, we postulate a model in which distinct cell types differentially use Ifit1 to inhibit infection of WNV-E218A, and likely other viruses, lacking 2′-*O* methylation. However, the viral NS5 2′-*O* methyltransferase enzyme also effectively antagonizes this Ifit1-independent pathway. Studies are underway to define additional ISGs (e.g., *Ifit2*, *Ifit3* or others) that explain Ifit1-independent control of WNV-E218A *in vivo*, and the precise cellular and biochemical antiviral mechanism(s) of action.

## Materials and Methods

### Ethics statement

This study was carried out in strict accordance with the recommendations in the Guide for the Care and Use of Laboratory Animals of the National Institutes of Health. The protocol was approved by the Institutional Animal Care and Use Committee at the Washington University School of Medicine (Assurance Number: A3381-01). All inoculation and experimental manipulation was performed under anesthesia that was induced and maintained with ketamine hydrochloride and xylazine, and all efforts were made to minimize suffering.

### Virus propagation and titration

WNV-WT and WNV-E218A were generated from an infectious cDNA clone of the New York 1999 strain and propagated in C6/36 *Aedes albopictus* or BHK21-15 cells as described [14], [54]. BHK21-15 and Vero cells were used to measure viral titers of infected cells or tissues by plaque or focus-forming assays [55], [56]. Viremia was determined by measuring viral RNA levels in serum using quantitative real-time reverse transcriptase polymerase chain reaction (qRT-PCR) and previously defined primer sets [55].

### Mouse experiments and tissue preparation

Wild type C57BL/6 mice were obtained commercially (Jackson Laboratories, Bar Harbor, ME). Congenic *Ifit1^−/−^* mice were generated from C57BL/6 embryonic stem cells that were produced by the trans-NIH Knock-Out Mouse Project and obtained from their repository (www.komp.org). *Ifit1* ^−/−^ ES cells were microinjected into (Cg)-*Tyr* ^c-2J^/J albino C57BL/6 mice recipient female mice. Chimeric mice with black coat color were selected and bred to wild type C57BL/6. Homozygous *Ifit1* ^−/−^ mice were generated by intercrossing the heterozygous animals and confirmed by PCR. *Ifit1^−/−^* mice were fertile and exhibited normal Mendelian frequencies.

Eight to ten week-old age-matched wild type or *Ifit1^−/−^* mice were inoculated with specified doses of WNV-WT or WNV-E218A diluted in Hanks balanced salt solution (HBSS) supplemented with 1% heat-inactivated fetal bovine serum (FBS) either by footpad (10^2^ to 10^5^ PFU in 50 µl) or intracranial injection (10^1^ to 10^5^ PFU in 10 µl). On specific days after infection, mice were sacrificed and extensively perfused with iced phosphate buffered saline (PBS), and organs were harvested, weighed, and stored at −80°C until further processing. Alternatively, groups of mice were monitored for twenty-one days after infection for survival.

### Primary cell infection

Primary bone marrow-derived DC, MEF, CN and GCN from wild type and *Ifit1^−/−^* mice were generated as described [57], [58]. Primary BMEC were prepared as described [59], [60] with some modifications. Eight week-old *Ifit1^−/−^* and congenic wild type C57BL/6 mice were sacrificed followed by dissection of cerebral cortices and removal of meninges. Cerebral cortical tissue was digested in 1 mg/ml type 2 collagenase (Worthington) and 30 U/ml DNase (Sigma-Aldrich) with shaking for 60 minutes at 37°C. Digested blood vessels were separated from myelinated cortical cells by centrifugation in a 20% BSA solution in DMEM (1,000× g) for 20 minutes. Isolated vessels were digested in 1 mg/ml collagenase-dispase (Roche) and 10 U/ml DNase with shaking for 45 minutes at 37°C. BMEC were then isolated by centrifugation on a 33% continuous Percoll gradient with 3% FBS (1,000× g) for 10 minutes, washed twice with DMEM, and plated in T-25 flasks coated with 0.1 mg/ml mouse collagen type IV (Sigma-Aldrich) and 0.1 mg/ml human fibronectin (Sigma-Aldrich). Multi-step virus growth curves were performed after infection at a multiplicity of infection (MOI) of 0.01. Cell viability of virus-infected cells was measured using Cell Titer-Glo Luminescent Cell Viability assay (Promega) according to the manufacturer's instructions.

### CNS leukocyte isolation and phenotyping

Quantification of infiltrating CNS lymphocytes was based on a published protocol [61]. Briefly, wild type and *Ifit1* ^−/−^ mouse brains were harvested on day 6 after intracranial inoculation, minced, and digested with 0.05% collagenase D, 0.1 µg/ml trypsin inhibitor TLCK, and 10 µg/ml DNase I in HBSS supplemented with 10 mM HEPES, pH 7.4 (Life Technologies). Cells were dispersed into single cell suspensions with a 70 µm cell strainer and centrifuged through a 37% Percoll cushion for 30 minutes (850× g at 4°C). Cells were counted and incubated with directly conjugated antibodies against CD3, CD4, CD8, CD45, and CD11b (BD Pharmingen) or D^b^-NS4B tetramer (NIH tetramer core facility). Alternatively, cells were incubated with 5 µg/ml brefeldin A (Sigma) for 6 h at 37°C with 10^−6^ M of immunodominant T cell peptides (D^b^-restricted NS4B 2488–2496 (SSVWNATTA) [36]. Cells were then washed, fixed, and permeabilized with FixPerm Buffer (eBioscience), and stained intracellularly for anti-IFN-γ (XMG1.2, BD Pharmingen), anti-TNF-α (MP6-XT22, eBioscience), or anti-granzyme B (GB12, Invitrogen). Lymphocytes were processed on a Canto II (BD Bioscience) using FACSDiva 6.1.1 software (BD Bioscience) and analyzed with FlowJo (Treestar).

### Depletion of CD8^+^ T cells

Mice were injected via an intraperitoneal route with 450 µg of either an anti-CD8-β chain specific monoclonal antibody (H35, rat IgG2b) or an isotype control (Jackson ImmunoResearch Laboratories). On the following day, mice were challenged via an intracranial route with 10^5^ PFU of WNV-E218A and followed for survival. Depletion was confirmed three days after administration of the antibodies by analysis of peripheral blood cells using flow cytometry.

### Quantification of mRNA levels by RT-PCR

Total RNA was isolated from primary cells by using the RNeasy kit according to the manufacturer's instructions (Qiagen). Following DNase I treatment (Invitrogen), cDNA was synthesized using oligo(dT) random hexamers, and Multiscribe reverse transcriptase (Applied Biosytems). Reverse transcription was performed using the following conditions: 25°C for 10 minutes, 48°C for 30 minutes, and 95°C for 5 minutes. The following primers were used to amplify murine *Ifit1*, *Ifit2*, and *GAPDH* mRNA: *Ifit1*, forward primer, 5′- GCCCTCAGCAGCACATCTTGCCAA -3′, reverse primer, 5′- CCTGCCTTCTGGGCTGCCTGTT -3′; Ifit2, forward primer, 5′- AAGGACCCGAAGAACCCAGAATTCAC -3′, reverse primer, 5′- GCCGGGTACCACATCACTAGTATTCAG -3′; and *GAPDH*, forward primer, 5′-GGCAAATTCAACGGCACAGT-3′, reverse primer, 5′-AGATGGTGATGGGCTTCCC-3′. PCR was performed using Platinum Taq master mix (Invitrogen) after 25 cycles at 65°C for 15 seconds and 72°C for 30 seconds. PCR products were analyzed using 2% agarose TAE gel, and imaged using AlphaEase FC software (Version 3.2.1, Alpha Innotech).

### Western blots

Primary cells were lysed in Laemmli buffer (BioRad). Samples were electrophoresed on 4 to 12% SDS-polyacrylamide gels (Invitrogen). Following transfer, nitrocellulose membranes were blocked with 5% nonfat dried milk overnight at 4°C. Membranes were probed with the rabbit anti-mouse Ifit1 ([7]) and β-actin (Abcam). Blots were incubated with peroxidase-conjugated secondary antibodies (Pierce) and visualized using ECL-Plus Immunoblotting reagents (Amersham Biosciences).

### Immunohistochemistry and confocal microscopy

Mice were infected with 10^5^ PFU of WNV-E218A via an intracranial route and sacrificed at day 6 after infection. Following sequential perfusion with 20 ml PBS and 20 ml 4% paraformaldehyde (PFA) in PBS, brains were harvested and fixed in 4% PFA in PBS overnight at 4°C. Tissues were cryoprotected in 30% sucrose, embedded, and frozen sections were cut. Tissue preparation and staining was performed as previously described [57], [61]. After saturating non-specific binding sites and permeabilizing cells, sections were incubated overnight at 4°C with anti-MAP2 (Chemicon) or anti-WNV (hyperimmune rat sera [62]). Primary antibodies were detected with secondary Alexa 488- or Alexa 555-conjugated goat anti-mouse or rat IgG (Molecular Probes). Nuclei were counter-stained with To-Pro3 (Molecular Probes). Terminal deoxynucleotidyltransferase-mediated dUTP-biotin nick end labeling (TUNEL) staining was performed using an *in situ* cell death detection kit (Roche) according to the manufacturer's instructions, with some modifications [38]. Fluorescence staining was visualized and quantitated with a Zeiss 510 Meta LSM confocal microscope.

### Statistical analysis

All data were analyzed using Prism software (GraphPad Prism4). An unpaired, two-tailed *t*-test was used to determine statistically significant differences for in vitro experiments. The Mann-Whitney test was used to analyze differences in viral burden. Kaplan-Meier survival curves were analyzed by the log rank test.

## Supporting Information

Figure S1 **Generation of** ***Ifit1^−/−^*** **mice.** **A**. Gene targeting strategy for genomic deletion of complete protein-encoding regions of *Ifit1* in embryonic stem cells. The C57BL/6 embryonic stem cells were produced by the trans-NIH Knock-Out Mouse Project, obtained from their repository (www.komp.org), and microinjected into (Cg)-*Tyr* ^c-2J^/J albino C57BL/6 mice recipient female mice. **B**. A representative image of a gel depicting PCR products from wild type, heterozygous and *Ifit1^−/−^* mouse tail DNA.
(TIF)

Figure S2 **Expression of Ifit1 in primary cells.** MEF and CN were generated from embryos, GCN were generated from neonates, macrophages and dendritic cells were derived from bone marrow, and BMEC were derived from adult wild type and *Ifit1^−/−^* mice. Primary cells were either mock-treated or treated with 100 IU of IFN-β for 24 hours, and cell lysates were harvested for RNA or protein analysis. (**A**) cDNA was generated from total RNA, PCR performed for *Ifit1*, *Ifit2*, and GAPDH, and products were resolved by 2% agarose gel electrophoresis. (**B**) Equivalent amounts of protein were loaded and separated on a 4–12% PAGE, transferred to nitrocellulose and blotted for Ifit1 or β-Actin.
(TIF)

Figure S3 **Cytokine responses in the serum of WNV-infected WT and** ***Ifit1^−/−^*** **mice.** Nine week-old wild type and *ifit1^−/−^* mice were inoculated subcutaneously with 10^5^ PFU of WNV-WT or WNV-E218A. At the indicated times after infection, serum was harvested and analyzed by Bioplex for IFN-γ, IL-1β, IL-6 and TNF-α. Data is shown as the concentration of cytokine per mL of serum for 9 to 11 mice per time point. Error bars represent standard error of the mean and dotted lines indicate the limit of detection of the assays. On days 3 and 4 after infection, IFN-γ and IL-6 levels were at or below the level of assay detection in wild type and *ifit1^−/−^* mice.
(TIF)

Text S1 **Supplemental methods.**
(DOCX)
