# Supplementary figures and images for: 2′-O Methylation of the Viral mRNA Cap by West Nile Virus Evades Ifit1-Dependent and -Independent Mechanisms of Host Restriction In Vivo
Source: PLoS Pathog. 2012 May 10;8(5):e1002698. doi: 10.1371/journal.ppat.1002698 (PMC3349756; doi:10.1371/journal.ppat.1002698)

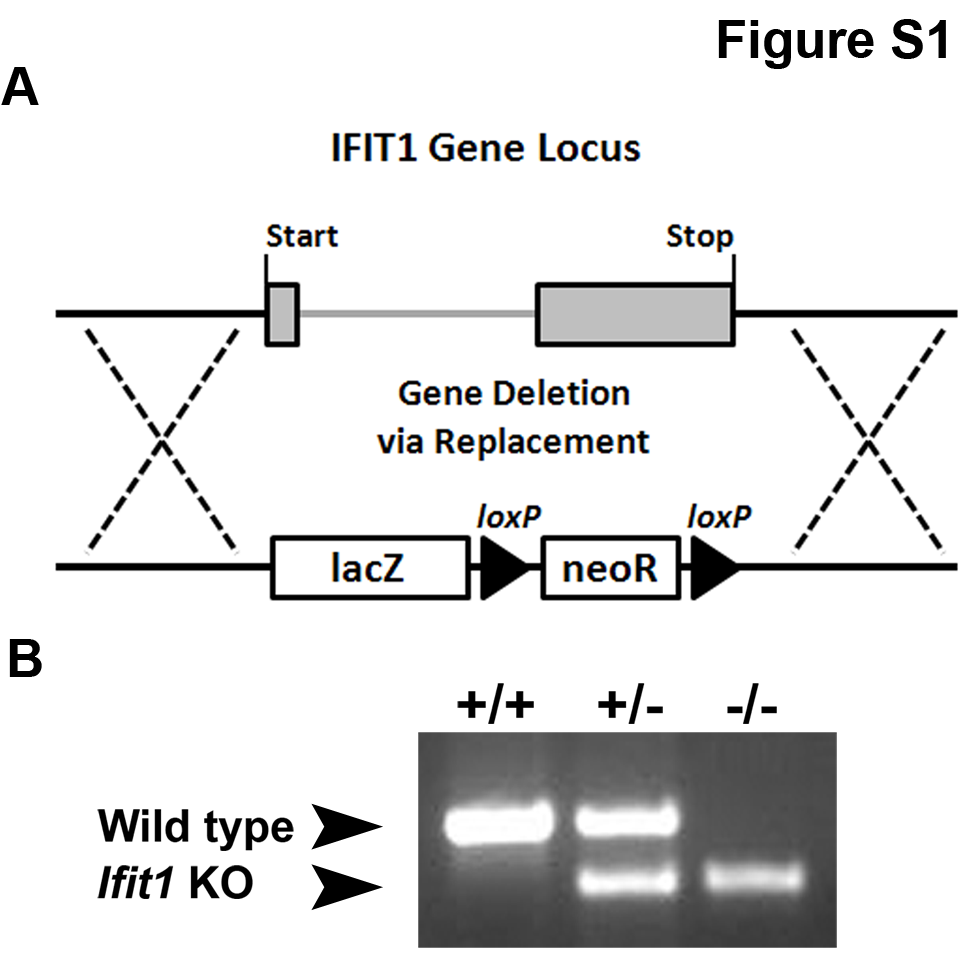

Supplement: Figure S1 — Generation of Ifit1−/− mice. A. Gene targeting strategy for genomic deletion of complete protein-encoding regions of Ifit1 in embryonic stem cells. The C57BL/6 embryonic stem cells were produced by the trans-NIH Knock-Out Mouse Project, obtained from their repository (www.komp.org), and microinjected into (Cg)-Tyr c-2J/J albino C57BL/6 mice recipient female mice. B. A representative image of a gel depicting PCR products from wild type, heterozygous and Ifit1−/− mouse tail DNA. (TIF) [file ppat.1002698.s001.tif]

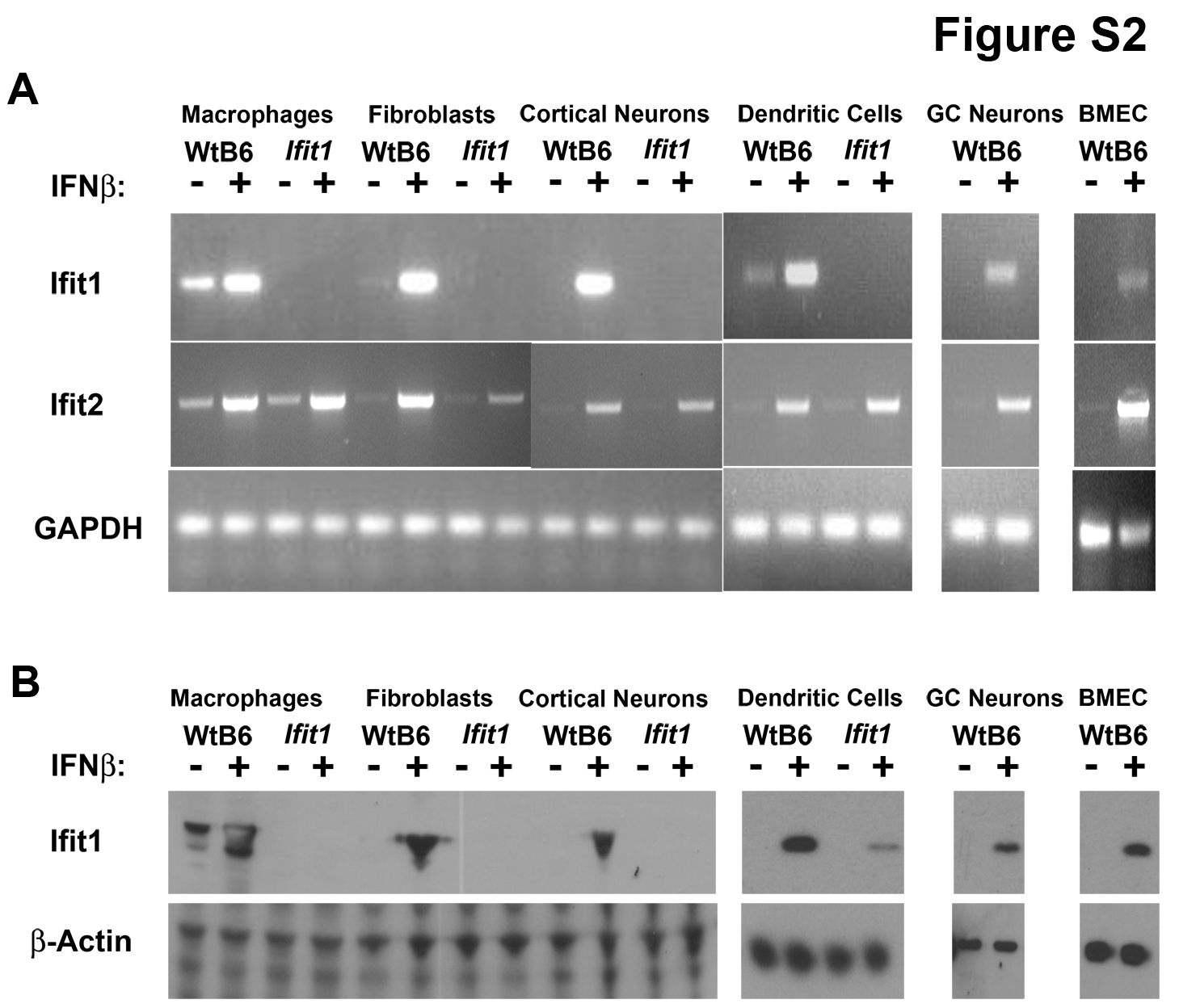

Supplement: Figure S2 — Expression of Ifit1 in primary cells. MEF and CN were generated from embryos, GCN were generated from neonates, macrophages and dendritic cells were derived from bone marrow, and BMEC were derived from adult wild type and Ifit1−/− mice. Primary cells were either mock-treated or treated with 100 IU of IFN-β for 24 hours, and cell lysates were harvested for RNA or protein analysis. (A) cDNA was generated from total RNA, PCR performed for Ifit1, Ifit2, and GAPDH, and products were resolved by 2% agarose gel electrophoresis. (B) Equivalent amounts of protein were loaded and separated on a 4–12% PAGE, transferred to nitrocellulose and blotted for Ifit1 or β-Actin. (TIF) [file ppat.1002698.s002.tif]

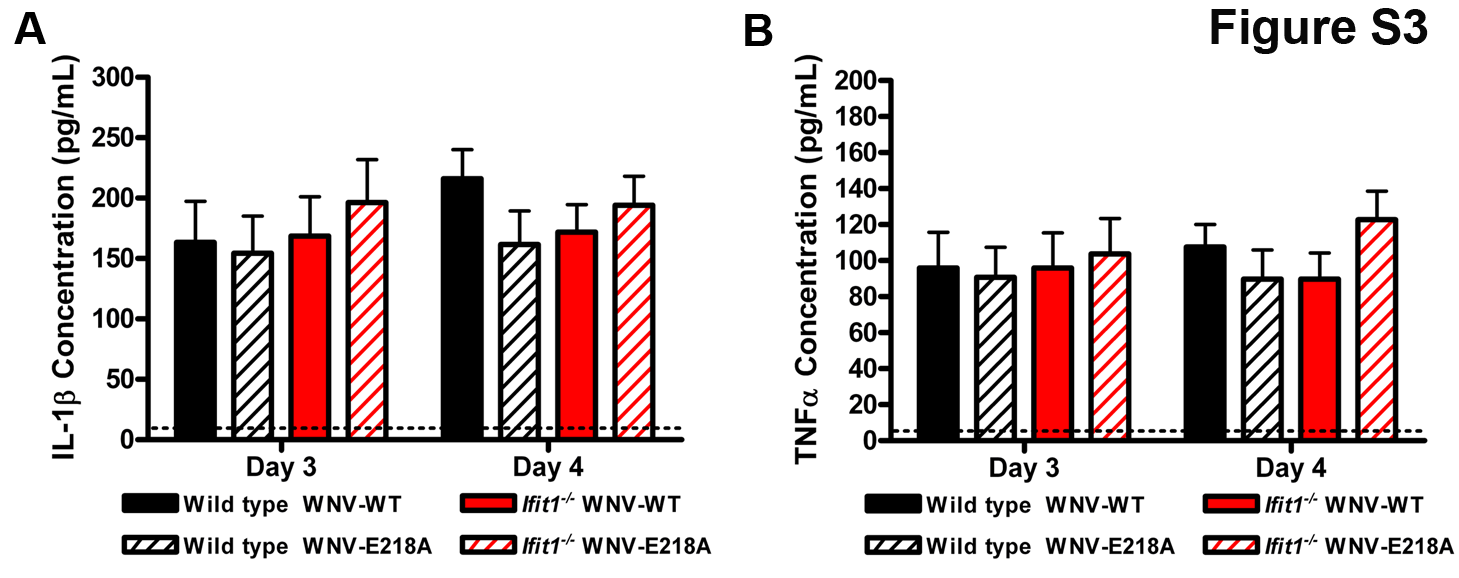

Supplement: Figure S3 — Cytokine responses in the serum of WNV-infected WT and Ifit1−/− mice. Nine week-old wild type and ifit1−/− mice were inoculated subcutaneously with 105 PFU of WNV-WT or WNV-E218A. At the indicated times after infection, serum was harvested and analyzed by Bioplex for IFN-γ, IL-1β, IL-6 and TNF-α. Data is shown as the concentration of cytokine per mL of serum for 9 to 11 mice per time point. Error bars represent standard error of the mean and dotted lines indicate the limit of detection of the assays. On days 3 and 4 after infection, IFN-γ and IL-6 levels were at or below the level of assay detection in wild type and ifit1−/− mice. (TIF) [file ppat.1002698.s003.tif]
