# Supplementary material for: 2′-O Methylation of the Viral mRNA Cap by West Nile Virus Evades Ifit1-Dependent and -Independent Mechanisms of Host Restriction In Vivo
Source: PLoS Pathog. 2012 May 10;8(5):e1002698. doi: 10.1371/journal.ppat.1002698 (PMC3349756; doi:10.1371/journal.ppat.1002698)
Supplement: Text S1 — Supplemental methods. (DOCX) [file ppat.1002698.s004.docx]

**Supplemental Methods**

**Quantification of serum cytokines.** Wild type and *ifit1^-/-^* mice were infected subcutaneously with 10^5^ PFU of WNV-WT and WNV-E218A, and on days 3 and 4 after infection serum was harvested and stored at -80°C until processing. Samples were diluted 1:4 in serum diluent (BioRad) and analyzed using a Bioplex cytokine bead array (BioRad) according to manufacturer’s protocols.
